# Changes in suicide attempt, suicidal ideation, and self-harm among Indian adolescents: comparison of cross-sectional surveys before (2016) and after (2023) COVID-19 pandemic

**DOI:** 10.1186/s13034-026-01041-4

**Published:** 2026-02-13

**Authors:** Samir Kumar Praharaj, Xiao Zhang, Praveen Arahanthabailu, Avinash G. Kamath, Rashmi Vishwanath, Anne Abio, Suvina Soans, Sneha Sesha, Vidisha Lahiri, Abel Buskutty, C. K. Shiva Prasad, Andre Sourander

**Affiliations:** 1 Present Address: Department of Psychiatry, Kasturba Medical College, Manipal Academy of Higher Education, Manipal, Karnataka 576104 India; 2 Research Centre for Child Psychiatry, University of Turku, Turku, Finland; 3 INVEST Research Flagship, University of Turku, Turku, Finland; 4 Manipal Hospital, Ambedkar Circle, Mangalore, Karnataka India; 5 Child and Adolescent Mental Health Services, Sandwell, Black Country Healthcare NHS Foundation Trust, West Bromwich, B708NY UK; 6 Father Muller Medical College, Mangalore, Karnataka India; 7 Chetna Psychiatric Hospital, Hyderabad, India; 8 Ishanya India Foundation, Bangalore, India; 9 Department of Child Psychiatry, Turku University Hospital, Turku, Finland

**Keywords:** Self-harm, Suicidal ideation, Suicide attempt, Adolescent, Pandemic, Temporal trend

## Abstract

**Background:**

This study examined trends in suicide attempts, suicidal ideation, and self-harm among adolescents before and after the COVID-19 pandemic, and explored their associations with psychopathology, bullying victimization, and perceived school safety.

**Methods:**

Two cross-sectional surveys were conducted in 2016 (*n* = 1,459) and 2023 (*n* = 1,153) among students in grades 7 to 9 (ages 11–17) across nine schools in South India. Suicidal behaviours were assessed using self-report items on suicide attempts, suicidal ideation, and self-harm. Risk factors included mental health (measured using the Strengths and Difficulties Questionnaire), traditional and cyberbullying victimization, and perceived school safety.

**Results:**

Between 2016 and 2023, the odds of suicide attempts increased from 2.1% to 6.6% (adjusted odds ratio, aOR = 3.45; 2.11–5.64), suicidal ideation from 4.7% to 11.6% (aOR = 2.64; 1.87–3.74), and self-harm from 8.5% to 15.2% (aOR = 1.65; 1.25–2.17). The largest increase in suicide attempts was observed among boys (aOR = 4.25; 2.05–8.80). Higher odds of suicidality were associated with emotional and conduct problems, physical health issues, bullying (traditional and cyber), male gender, urban residence, and non-nuclear family structures. Feeling safe at school and prosocial behaviour were protective factors.

**Conclusion:**

Rates of adolescent suicidality increased after the pandemic. Risk factors identified were bullying, mental and physical health issues, and family structure, while school safety and prosocial behaviour are protective. Interventions targeting these areas are urgently needed to mitigate rising suicide risk among adolescents.

**Supplementary Information:**

The online version contains supplementary material available at 10.1186/s13034-026-01041-4.

## Introduction

Suicide is a major global public health concern among adolescents, with 3.8 deaths per 100,000 people across all ages [1]. India has approximately 253 million adolescents, representing 20.9% of its total population (Census of India, 2011). Worldwide, 14–23% of youth report suicidal ideation, 4–24% make a plan, and 5–16% attempt suicide [2]). Suicidal behaviour follows a continuum—from ideation to attempts and completion [3]. Suicidal ideation raises the risk of attempts and suicide, but its transient nature complicates risk identification. While rates are rising in Asia, including India, they are declining in Europe [4, 5]. A review found 11% of Indian youth report ideation and 3% report plans or attempts [6]. Suicidality is consistently linked to mental health issues [7], bullying [8, 9], and feeling unsafe at school [10], alongside other contributing factors such as academic stress, substance use, interpersonal difficulties, and financial problems.

Self-harm affects around 17% of adolescents globally and usually declines with age [11, 12]. High rates are also seen in Indian adolescents, though patterns may differ from Western contexts [13]. The relationship between self-harm and suicide is complex—some escalate to suicide attempts, increasing the risk of death [14, 15], while others engage in non-suicidal self-injury (NSSI), which carries lower suicidal risk but may still result in accidental fatalities [12, 16]. Regardless of intent, self-harming adolescents are vulnerable. Adolescent males have higher suicide completion rates, but suicidal ideation and non-fatal attempts are more common among females [1, 2].

The COVID-19 pandemic worsened adolescent mental health globally, increasing suicidality and self-harm [17, 18]. In India, it was similarly linked to rising adolescent suicidal behaviour [19]. The pandemic served as a natural experiment to study changes in suicidality [20, 21], yet no Indian study has compared before and after pandemic trends. Few Asian studies have done so—for example, adolescent suicidality in South Korea, once declining, rose post-pandemic [22], while in China, suicidal ideation initially spiked but later declined [23].

This is the first epidemiological study from India to examine temporal trends in adolescent suicidality and self-harm. The first aim is to compare the prevalence of suicide attempts, suicidal ideation, and self-harm before (2016) and after (2023) the COVID-19 pandemic. Based on global findings, we hypothesize that rates of suicidal behaviour will be higher after the pandemic. The second aim is to examine associations between suicidality and mental health problems, bullying victimization, and school safety. We hypothesize that adolescents reporting suicidal behaviour will also report greater mental health difficulties, higher exposure to bullying, and feeling unsafe at school.

## Methods

### Study setting and participants

**Fig. 1:**
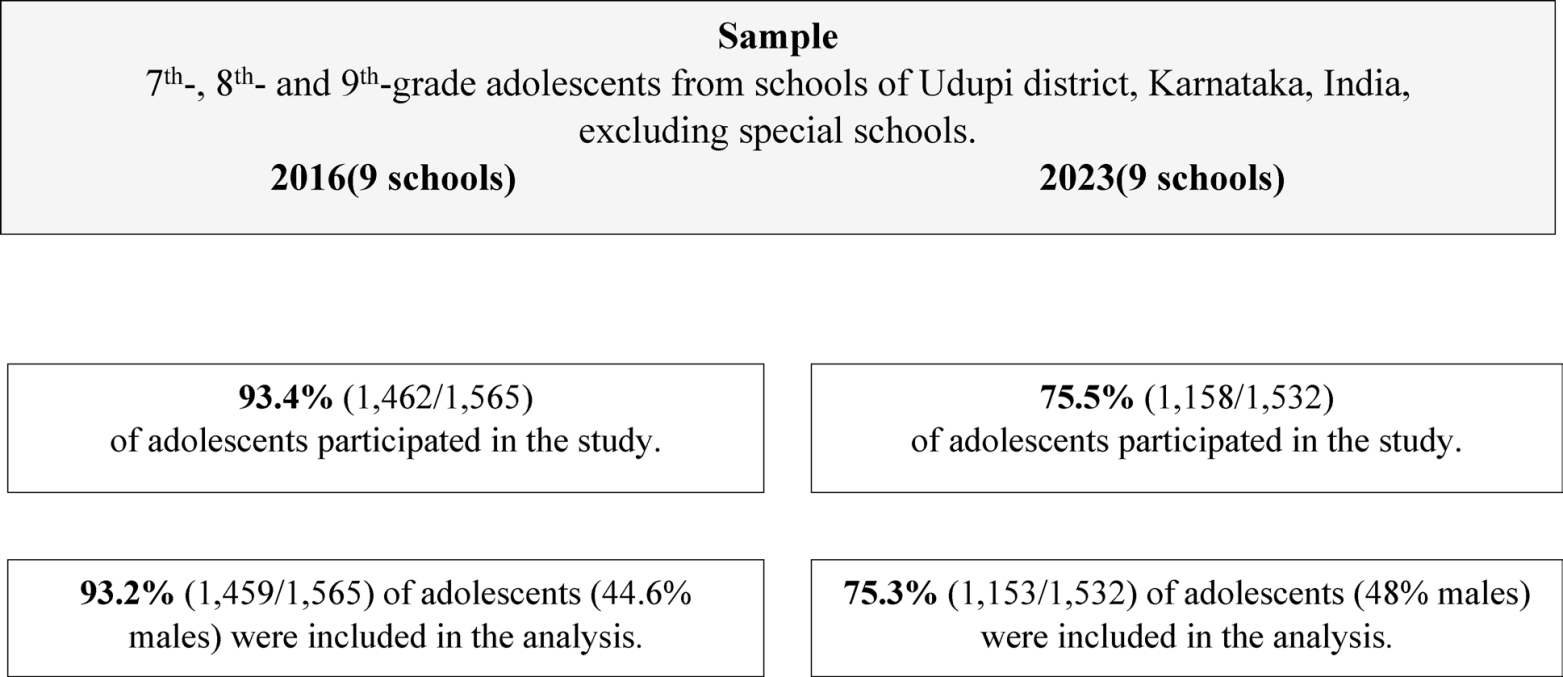
Flow of participants across two surveys (2016 and 2023)

This study was part of the Global Child and Adolescent Mental Health Study (GCAMHS), a cross-cultural investigation conducted across several Asian and European countries. Two surveys were conducted at the Department of Psychiatry, Kasturba Medical College, Manipal—a tertiary care center in South India—before the COVID-19 pandemic (July–Nov 2016) and after (Oct 2023–Feb 2024). Both involved students from the same schools in Udupi district, Karnataka, although they examined distinct populations (see Fig. 1). Udupi, a coastal district with both rural and urban areas, is representative of typical Indian districts, though distinct from major metros like Bengaluru. Participants were students in grades seven to nine, aged 11–17 years.

### Ethical information

Permission to conduct the study was obtained from Block Education Officer and school principals. Ethical approval was granted by Institutional Ethics Committee of Kasturba Medical College and Kasturba Hospital (IEC 782/2015, dated 8 Dec 2015; IEC 342/2023, dated 13 Sept 2023). Written informed consent was obtained from parents and assent from all participating students, in accordance with ethics guidelines.

### Measures

*Suicidality* was assessed using three questions on self-harm (“Have you intentionally hurt yourself for example by cutting or burning your skin?”), suicidal ideation (“Have you thought seriously about committing suicide?”), and suicide attempts (“Have you tried committing suicide?”). Participants reflected on the past six months and responded with “No, I have not,” “Yes, once,” or “Yes, more than once.” For analysis, responses were dichotomized into “No” and “Yes.”

*Mental health* was assessed using the 25-item Strengths and Difficulties Questionnaire (SDQ) [24], which measures internalizing and externalizing problems across five subscales: emotional symptoms, conduct problems, hyperactivity, peer problems, and prosocial behavior. Each subscale has five items scored 0–2 on a three-point scale (“not true,” “somewhat true,” “certainly true”) reflecting feelings over the past six months. Five positively worded items were reverse scored. Higher scores indicate more problems, except for prosocial behavior. SDQ has been used in previous Indian studies [25, 26]. Cronbach’s α in our sample was 0.65 overall, 0.67 for girls, and 0.68 for boys.

*Traditional bullying victimization* over past six months was assessed using two questions: “How often have you been bullied in school in the past six months?” and “How often have you been bullied outside of school in the past six months?” *Cyberbullying victimization* was assessed with the question, “During the past six months, how often have you been cyberbullied?” Standard definitions of bullying were provided. Response options (“never,” “less than once a week,” “more than once a week,” and “almost every day”) were collapsed into three categories for analysis, combining the two most frequent. *School experience* was measured with the question “I feel safe at school,” with response options of “never,” “sometimes,” “often,” and “always.”

*Demographic information* included age; gender (boy/girl); residence (rural/urban); family structure (both biological parents, single parent, step-parent, or other relatives); place of birth (within or outside Karnataka); native language (Kannada, Tulu, Konkani, or other). Physical health was assessed with the question: “Do you have an illness, disability, or other health-related problem?”

### Procedure

Both public and private schools were included in the study. Data were collected with the help of school teachers during a pre-scheduled period when regular classes were cancelled [27]. Consent forms and information sheets were distributed in advance, and only students with written parental consent were invited to participate. Assent from the participants was also obtained. On the day of data collection, all present students were approached. Questionnaires were completed anonymously in under an hour, with researchers and teachers available to answer questions. Completed forms were collected at the end of the session. Participants were informed about available mental health services if they reported suicidal thoughts or other mental health concerns.

### Statistical analysis

Data were analyzed using Stata version 18 (StataCorp, TX, USA). Pearson’s chi-squared tests compared proportions across time points. Logistic regression was used to report the prevalence of self-harm, suicidal ideation, and suicide attempts, with unadjusted and adjusted odds ratios (OR, aOR) and 95% confidence intervals (CIs), using 2016 as the reference year. Individual-level prevalence of *self-harm only*, *suicidal ideation only*, *suicidal ideation AND self-harm*, and *suicide attempts* was reported. Univariate and multivariable binary logistic regression identified risk factors for suicidality. Multiple linear regression was used for SDQ domain scores. P-values < 0.05 were considered statistically significant.

## Results

### Demographics

Complete data were available for 1,459 participants in 2016 (93.4%) and 1,153 in 2023 (75.5%). Demographic comparisons are shown in Table 1. In 2023, a significantly higher proportion of students were over 13 years old (*P* < .001), and fewer were born outside Karnataka (*P* < .001). While Kannada was the predominant native language in 2016, there was greater representation of Tulu and other languages in 2023 (*P* < .001). The 2016 sample also had more children with at least one parent born outside Karnataka (*P* < .001).

**Table 1:** Sample characteristics

Characteristics	2016 (*n* = 1459)	2023 (*n* = 1153)	*p*-value^†^
	*n *(%)	*n *(%)	
Gender			0.075
Females	809(55.5)	599(52)	
Males	650(44.6)	554(48)	
Age			
≤ 13	858(58.8)	534(46.3)	**< 0.001**
> 13	601(41.2)	619(53.7)	
Place of residence			0.877
Urban	1,140(78.1)	898(77.9)	
Rural	319(21.9)	255(22.1)	
Place of birth			**< 0.001**
Karnataka	1,405(96.4)	1,140(99.2)	
Outside of Karnataka	53(3.6)	9(0.8)	
Native language			**< 0.001**
Kannada	838(59)	358(31.3)	
Konkani	145(10.2)	153(13.4)	
Tulu	289(20.4)	474(41.4)	
Other	148(10.4)	159(13.9)	
Parents background*			**< 0.001**
Both from Karnataka	1,312(93.6)	1,143(99.5)	
Only one from Karnataka	59(4.2)	4(0.4)	
Both not from Karnataka	31(2.2)	2(0.2)	
Family structure			0.234
Two biological parents	1,376(94.6)	1,052(93.4)	
One biological parent	58(4)	49(4.4)	
Other	21(1.4)	26(2.3)	
Family structure+			**< 0.001**
Nuclear family	1,375(94.4)	956(84.6)	
Extended family	0	93(8.2)	
Single parent	58(4)	39(3.5)	
Only relatives	14(1)	19(1.7)	
Biological parents and stepparent	0	4(0.4)	
Adoptive	4(0.3)	1(0.1)	
Other	5(0.3)	18(1.6)	
Perceived family wealth			-
Less than normal	-	105(10)	
Normal	-	371(35.4)	
More than normal	-	573(54.6)	

^†^Chi-squared test

*Parents’ place of birth

Significant P values are bolded

### Prevalence of suicidal behavior

**Fig. 2:**
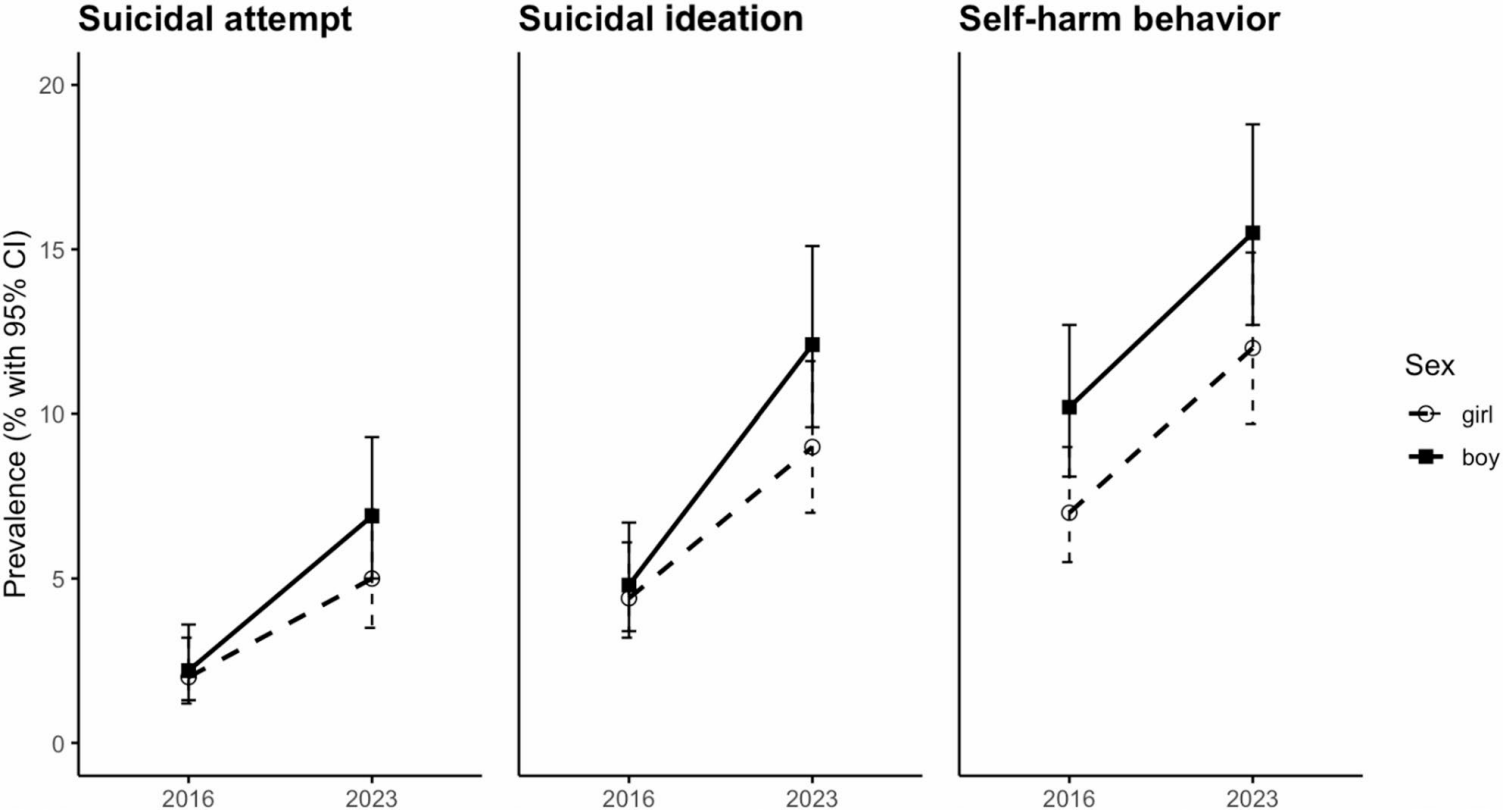
Temporal trends (2016 to 2023) in prevalence of suicide attempts, suicidal ideation, and self-harm, stratified by gender

Table 2 shows prevalence, unadjusted OR, and aOR with 95% CIs for suicide attempts, suicidal ideation, and self-harm, for total sample and by gender. From 2016 to 2023, prevalence increased for suicide attempts (2.1% to 6.6%; aOR = 3.45; 2.11–5.64), suicidal ideation (4.7% to 11.6%; aOR = 2.64; 1.87–3.74), and self-harm (8.5% to 15.2%; aOR = 1.65; 1.25–2.17). Increases were also seen by gender, with the largest rise in suicide attempts among girls (2% to 5.6%; aOR = 3.01; 1.52–5.95) and boys (2.2% to 7.6%; aOR = 4.25; 2.05–8.80). A significant interaction was found between year and age for self-harm (*P* = .018), and between gender and age for suicidal thoughts (*P* = .03); other interactions were not significant. Figure 2 shows temporal trends in prevalence of suicide attempts, suicidal ideation, and self-harm, stratified by gender.

**Table 2:** Prevalence, unadjusted and adjusted ORs comparing years (for total sample, girls, and boys)

	2016 *n *(%)	2023 *n *(%)	Unadjusted OR (95% CI)	Adjusted OR^†^(95% CI)
**ToTal**				
*Prevalence*				
Self-harm	123(8.5)	158(15.2)	1.92(1.49–2.46)***	1.65(1.25–2.17)***
Suicidal ideation	67(4.7)	121(11.6)	2.67(1.96–3.65)***	2.64(1.87–3.74)***
Suicide attempt	30(2.1)	68(6.6)	3.26(2.11–5.05)***	3.45(2.11–5.64)***
*Individual level*				
No suicidality	1262(88.3)	807(77.9)	1	
Self-harm only	87(6.1)	90(8.7)	1.62(1.19–2.20)**	1.29(0.92–1.80)
Suicidal ideation only	36(2.5)	46(4.4)	2.00(1.28–3.12)**	1.92(1.17–3.14)*
Suicidal ideation AND self-harm	15(1.1)	25(2.4)	2.61(1.37–4.97)**	2.46(1.21–4.99)*
Suicide attempt	30(2.1)	68(6.6)	3.54(2.29–5.50)***	3.68(2.25–6.03)***
**Girl**				
*Prevalence*				
Self-harm	57(7.1)	72(13.5)	2.03(1.41–2.93)***	1.87(1.25–2.82)**
Suicidal ideation	36(4.5)	54(10.1)	2.36(1.52–3.65)***	2.61(1.59–4.26)***
Suicide attempt	16(2.0)	30(5.6)	2.89(1.56–5.35)**	3.01(1.52–5.95)**
*Individual level*				
No suicidality	715(89.9)	426(79.9)	1	
Self-harm only	39(4.9)	47(8.8)	2.02(1.30–3.14)**	1.69(1.03–2.77)*
Suicidal ideation only	19(2.4)	22(4.1)	1.94(1.04–3.63)*	2.03(1.00–4.11)*
Suicidal ideation AND self-harm	6(0.8)	8(1.5)	2.24(0.77–6.49)	2.80(0.87–9.00)
Suicide attempt	16(2.0)	30(5.6)	3.15(1.70–5.84)***	3.27(1.65–6.48)**
**Boy**				
*Prevalence*				
Self-harm	66(10.3)	86(17.0)	1.78(1.26–2.51)**	1.47(1.01–2.15)*
Suicidal ideation	31(4.9)	67(13.3)	2.98(1.91–4.64)***	2.85(1.73–4.68)***
Suicide attempt	14(2.2)	38(7.6)	3.61(1.93–6.74)***	4.25(2.05–8.80)**
*Individual level*				
No suicidality	547(86.1)	381(75.8)	1	
Self-harm only	48(7.6)	43(8.6)	1.29(0.84–1.98)	0.98(0.61–1.56)
Suicidal ideation only	17(2.7)	24(4.8)	2.03(1.07–3.82)*	1.96(0.97–3.97)
Suicidal ideation AND self-harm	9(1.4)	17(3.4)	2.71(1.20–6.15)*	2.36(0.97–5.75)
Suicide attempt	14(2.2)	38(7.6)	3.90(2.08–7.29)***	4.47(2.15–9.27)***

^†^Total sample is adjusted by sex, age, urbanity, place of birth, native language, parents’ place of birth, and family structure; gender different results are adjusted by age, urbanity, place of birth, native language, parents’ place of birth, and family structure

*p<.05

**p<.01

***p<.001

At the individual level, the odds of *suicide attempts* increased significantly from 2.1% to 6.6% (aOR = 3.68; 2.25–6.03). The odds of *suicidal ideation AND self-harm* rose from 1.1% to 2.4% (aOR = 2.46; 1.21–4.99) and *suicidal ideation only* increased from 2.5% to 4.4% (aOR = 1.92; 1.17–3.14). The odds of *self-harm only* increased from 6.1% to 8.7%, but this was not statistically significant. Among girls, the odds of *suicide attempts* (aOR = 3.27; 1.65–6.48), *suicidal ideation only* (aOR = 2.03; 1.00–4.11), and *self-harm only* (aOR = 1.69; 1.03–2.77) increased significantly, while the odds of *suicidal ideation AND self-harm* remained stable. Among boys, only *suicide attempts* showed a significant increase (aOR = 4.47; 2.15–9.27); other categories showed no significant change.

### Risk factors for suicidal behavior

Table 3 presents multivariable logistic regression results for risk factors of suicide attempts, suicidal ideation, and self-harm (univariate results are in Supplementary table **S1**). Time (i.e., 2023 vs. 2016) was significantly associated with increased odds of suicide attempts (OR = 2.82; 1.54–5.18) and suicidal ideation (OR = 1.95; 1.28–2.96), but not self-harm. No significant differences were found across gender or age groups for these outcomes.

**Table 3:** Multivariable logistic regression for risk factors

	Self-harm		Suicidal ideation		Suicide attempts	
	*n *(%)	OR (95% CI)	*n *(%)	OR (95% CI)	*n *(%)	OR (95% CI)
Year						
2016	123(8.54)	1	67(4.7)	1	30(2.1)	1
2023	158(15.2)	1.21(0.87–1.68)	121(12)	1.95(1.28–2.96)**	68(6.6)	2.82(1.54–5.18)**
Gender						
Females	129(9.66)	1	90(6.8)	1	46(3.5)	1
Males	152(13.3)	1.10(0.81–1.50)	98(8.6)	0.97(0.66–1.43)	52(4.6)	0.69(0.39–1.20)
Age						
≤ 13	145(10.8)	1	85(6.3)	1	42(3.1)	1
> 13	136(12)	1.08(0.80–1.45)	103(9.1)	1.33(0.92–1.92)	56(5)	1.58(0.94–2.66)
Place of residence						
Rural	47(8.29)	1	43(7.6)	1	23(4.1)	1
Urban	234(12.2)	1.77(1.22–2.58)**	145(7.6)	1.06(0.70–1.62)	75(4)	1.04(0.57–1.90)
Place of birth						
Outside of Karnataka	2(3.51)	1	3(5.3)	1	2(3.6)	1
Karnataka	278(11.5)	1.50(0.33–6.73)	185(7.7)	1.26(0.26–6.26)	95(4)	0.17(0.03–0.85)*
Native language						
Kannada	115(9.9)	1	83(7.2)	1	48(4.2)	1
Konkani	24(8.9)	0.71(0.41–1.24)	17(6.4)	0.73(0.37–1.42)	5(1.9)	0.22(0.07–0.76)*
Tulu	97(13.6)	1.30(0.91–1.85)	66(9.3)	1.09(0.70–1.67)	35(4.9)	0.62(0.34–1.14)
Other	44(15.3)	1.51(0.96–2.37)	22(7.7)	0.57(0.30–1.08)	10(3.5)	0.33(0.13–0.85)*
Parents background						
Both from Karnataka	268(11.5)	1	175(7.6)	1	93(4)	1
Only one from Karnataka	6(9.8)	1.18(0.45–3.09)	4(6.6)	1.42(0.43–4.68)	2(3.3)	1.23(0.22–6.77)
Both not from Karnataka	1(3.1)	1.00(empty)	4(13)	7.57(1.40–41.01)*	0	1.00(empty)
Family structure						
Two biological parents	251(10.9)	1	163(7.1)	1	83(3.6)	1
One biological parent	17(16.2)	1.02(0.51–2.03)	13(12)	1.29(0.59–2.83)	8(7.6)	1.07(0.38–3.06)
Other	8(17.8)	1.71(0.70–4.16)	7(16)	2.71(1.06–6.96)*	4(9.3)	2.82(0.82–9.74)
Traditional bullying in school						
Not at all	166(8.6)	1	104(5.4)	1	52(2.7)	1
Less than once a week	67(20.4)	1.57(1.07–2.31)*	39(12)	1.40(0.86–2.28)	20(6.2)	1.14(0.57–2.28)
More than once a week	42(26.1)	1.30(0.77–2.20)	42(27)	2.16(1.23–3.79)**	24(15)	1.46(0.68–3.13)
Traditional bullying outside of school						
Not at all	195(9)	1	128(5.9)	1	61(2.8)	1
Less than once a week	51(30.9)	2.39(1.50–3.78)***	36(22)	1.58(0.91–2.76)	23(14)	3.01(1.51–6.02)**
More than once a week	30(38)	2.67(1.44–4.97)**	22(29)	1.86(0.91–3.79)	13(17)	2.84(1.13–7.13)*
Cyberbullying						
Not at all	229(10.2)	1	148(6.6)	1	71(3.2)	1
Less than once a week	27(40.3)	2.94(1.62–5.35)***	21(31)	2.91(1.49–5.68)**	15(22)	4.35(1.98–9.51)***
More than once a week	13(28.3)	2.33(0.99–5.51)	10(22)	2.17(0.78–6.01)	9(20)	5.32(1.68–16.83)**
School safety						
Never feel safe	39(24.4)	1	29(19)	1	24(15)	1
Sometimes feel safe	71(16.1)	0.59(0.34–1.01)	48(11)	0.47(0.24–0.89)*	22(5)	0.29(0.13–0.66)**
Often feel safe	29(12.8)	0.60(0.32–1.12)	11(4.9)	0.28(0.12–0.66)**	6(2.7)	0.26(0.09–0.75)*
Always feel safe	137(8.5)	0.45(0.27–0.75)**	97(6)	0.45(0.25–0.83)*	44(2.8)	0.26(0.12–0.54)***
Health problems						
No	221(10.4)	1	141(6.7)	1	69(3.3)	1
Yes	52(16.9)	1.41(0.95–2.08)	39(13)	2.12(1.34–3.35)**	25(8.2)	2.93(1.59–5.39)**
SDQ^†^	Mean (SD)		Mean (SD)		Mean (SD)	
Emotion problems	2.9(2.2)	1.08(1.01–1.17)*	2.9(2.2)	1.20(1.10–1.31)***	2.9(2.2)	1.16(1.03–1.32)*
Hyperactivity	2.8(1.9)	1.06(0.97–1.16)	2.8(1.9)	1.08(0.98–1.20)	2.8(1.9)	0.95(0.82–1.10)
Conduct problems	2.8(1.7)	1.20(1.10–1.31)***	2.8(1.7)	1.26(1.13–1.40)***	2.8(1.7)	1.25(1.08–1.45)**
Peer problems	2.6(1.7)	1.01(0.92–1.10)	2.6(1.7)	0.98(0.88–1.09)	2.6(1.7)	1.01(0.87–1.17)
Prosocial behavior	7.4(2.2)	0.97(0.91–1.05)	7.4(2.2)	0.97(0.89–1.07)	7.4(2.2)	0.81(0.72–0.91)***

SDQ: Strengths and Difficulties Questionnaire

^†^Odds calculated for 1 SD change

*p<.05

**p<.01

***p<.001

The odds of suicide attempts increased (for one SD change) significantly with higher emotional problems (OR = 1.16; 1.03–1.32) and conduct problems (OR = 1.25; 1.08–1.45) and decreased with higher prosocial behaviour (OR = 0.81; 0.72–0.91). No significant associations were found for hyperactivity or peer problems. Similarly, the odds of suicidal ideation were significantly associated with emotional problems (OR = 1.20; 1.10–1.31) and conduct problems (OR = 1.26; 1.13–1.40), but not with hyperactivity or peer problems. The odds of self-harm were also significantly associated with emotional problems (OR = 1.08; 1.01–1.17) and conduct problems (OR = 1.20; 1.10–1.31), with no significant associations with hyperactivity or peer problems.

The odds of suicide attempts were significantly higher with infrequent (OR = 3.01; 1.51–6.02) and frequent (OR = 2.84; 1.13–7.13) traditional bullying outside of school, as well as infrequent (OR = 4.35; 1.98–9.51) and frequent cyberbullying (OR = 5.32; 1.68–16.83). The odds of suicidal ideation were significantly associated with frequent traditional bullying in school (OR = 2.16; 1.23–3.79) and infrequent cyberbullying (OR = 2.91; 1.49–5.68). The odds of self-harm were significantly associated with infrequent traditional bullying in school (OR = 1.57; 1.07–2.31), both infrequent (OR = 2.39; 1.50–3.78) and frequent (OR = 2.67; 1.44–4.97) traditional bullying outside of school, and infrequent cyberbullying (OR = 2.94; 1.62–5.35).

Adolescents who felt safe at school had significantly lower odds of suicide attempts—whether they felt safe *sometimes* (OR = 0.29; 0.13–0.66), *often* (OR = 0.26; 0.09–0.75), or *always* (OR = 0.26; 0.12–0.54). Similar reductions were seen for suicidal ideations: *sometimes* (OR = 0.47; 0.24–0.89), *often* (OR = 0.28; 0.12–0.66), or *always* (OR = 0.45; 0.25–0.83). The odds of self-harm were also significantly lower among students who *always* felt safe at school (OR = 0.45; 0.27–0.75). In contrast, adolescents with physical health problems had higher odds of suicide attempts (OR = 2.93; 1.59–5.39) and suicidal ideation (OR = 2.12; 1.34–3.35), with no significant association with self-harm.

Adolescents from urban areas had higher odds of self-harm than those from rural areas (OR = 1.77; 1.22–2.58). Those born within Karnataka had significantly lower odds of suicide attempts (OR = 0.17; 0.03–0.85), with no significant differences for self-harm or suicidal ideation. Compared to Kannada speakers, the odds of suicide attempts were lower among Konkani (OR = 0.22; 0.07–0.76) and other language speakers (OR = 0.33; 0.13–0.85). Suicidal ideation was significantly higher among adolescents whose parents were both from outside Karnataka (OR = 7.57; 1.40–41.01), and among those not living with both biological parents (OR = 2.71; 1.06–6.96).

## Discussion

This study is the first in India to examine temporal trends in adolescent suicidal behavior at two time points—2016 (before pandemic) and 2023 (after pandemic)—using data from the same schools, consistent methodology, and a validated questionnaire. School-based data collection during scheduled periods under teacher supervision ensured high response rates and completeness [27]. Providing information about available mental health services for students experiencing difficulties also helped build confidence among teachers and participants. Findings show increased odds of suicide attempts, suicidal ideation, and self-harm post-pandemic, with significant associations to mental health problems, bullying, and feeling unsafe at school.

### Temporal trends in suicidal behaviour and self-harm

#### Changes in suicidal attempts over time

The six-month odds of suicide attempts among adolescents increased 3.5 times from 2016 to 2023, consistent with global trends [4, 17, 22, 23]. A systematic review reported increased emergency department visits for suicide attempts during the pandemic [17]. Contributing factors include social isolation, increased screen time, reduced access to education and healthcare, increased family and financial stress [28]. While the pandemic played a significant role [17, 19, 22], contextual influences are important. For instance, in China, suicide attempts initially surged post-pandemic but later declined, reflecting local factors [23]. In India, rising adolescent suicide trends predated the pandemic [5, 29], and a qualitative study identified interpersonal events, often amid chronic stress, as common triggers for suicide attempts [30].

#### Changes in suicidal ideation over time

The odds of suicidal ideation rose 2.5 times from 2016 to 2023, with the proportion of adolescents experiencing suicidal ideation only (without self-harm or attempts) nearly doubling in both genders. Occasional suicidal ideas without accompanying self-harm or attempts may be common among healthy adolescents, often linked to developmental or existential concerns [31], but persistent and recurrent ideations are associated with higher risk of suicide attempts [32].

The odds of suicidal ideation with self-harm nearly doubled among boys but remained low and stable among girls. This co-occurrence is concerning, as self-harm significantly raises the risk of suicide attempts in adolescents with suicidal ideation [33]. Exposure to self-harm in family and friends further increases this risk [34]. Joiner’s *interpersonal theory* posits that suicidal ideation alone is necessary but insufficient for attempts; *acquired capabilities*—like overcoming fear of death and increased pain tolerance—are also needed [35, 36]. These capabilities are linked to *painful and provocative events* (PPE), including past self-harm [35, 37, 38]. The pandemic may have acted as a PPE, enhancing these capabilities and increasing suicide attempt risk [38]. However, these interpretations are theoretical, as the relevant constructs were not directly assessed.

#### Changes in self-harm over time

The odds of self-harm increased 1.5 times from 2016 to 2023, with a greater rise among girls than boys. Similar gender differences have been reported across cultures in a systematic review comparing before and after pandemic rates [28]. Among girls, the odds of self-harm without suicidal ideation nearly doubled, reaching levels comparable to after pandemic rates in boys. In contrast, self-harm without suicidal ideation among boys remained stable. A survey across six Asian countries found that Indian girls were more likely to report isolation, stress, and concerns about education and household income [39], which may explain the greater increase in self-harm among girls. Other countries have also reported higher pandemic-related stress among female adolescents globally [40–42].

The prevalence of suicidal ideation in our samples was 5% in 2016 and 11% in 2023, compared to a pooled prevalence of 11% among Indian adolescents reported in a recent systematic review [6]. These differences may reflect variations in sample characteristics, study design, and regional context. Similarly, differences in self-harm rates across studies may be due to varying definitions; studies including older adolescents (e.g. Sinha et al. [43]) or minor NSSI (e.g., Bhola et al. [44]) often report higher rates.

### Risk factors for suicidal behavior

#### Psychopathology

Emotional and conduct problems in adolescents were associated with increased odds of suicide attempts, suicidal ideation, and self-harm in our sample. This aligns with previous findings that mental health issues are major contributors to suicidality among Indian adolescents [45]. A recent meta-analysis confirms that both internalizing and externalizing symptoms predict suicidal behaviour among adolescents [7]. However, Commisso et al. [46] found that childhood externalizing problems, with or without co-occurring internalizing problems, were associated with an increased risk of suicide attempts, but not with suicidal ideation without attempts, highlighting the complexity of these relationships. In contrast, prosocial behaviour was associated with reduced risk of suicide attempts in our study, consistent with prior findings that suggest protective effects of empathy, helping behaviour, and social connectedness [47, 48]. Social support—through school, family, and peer relationships—serves as buffer against *thwarted belongingness* and *perceived burdensomeness* and acts as protective factor against suicidal ideation and attempts [38].

#### Bullying

Bullying victimization—including both traditional forms and cyberbullying—emerged as a significant risk factor for suicidality among adolescents, consistent with findings from studies worldwide [8, 9, 49–51]. This is particularly concerning given that nearly one-third of school children experience victimization in various studies [52–54]. Violence has also been identified as a risk factor in a scoping review of Indian studies [45]. The strong link between peer victimization and suicidal ideation and attempts may be explained by increased feelings of *thwarted belongingness* and an enhanced *acquired capability*, both of which raise the risk of suicidal ideation and attempts [35, 38].

The association between cyberbullying and suicide attempts was stronger than that of traditional bullying, highlighting the significance of this pervasive and widespread form of victimization [50]. Although less frequent, cyberbullying has a more severe impact [51, 53]. It can involve both peer and adult perpetrators, making it a stronger PPE than traditional bullying [38]. This increased severity may enhance the acquired capability, thereby raising the risk of suicide attempts, as outlined in the interpersonal theory of suicide [35, 38].

#### School safety

Feeling safe in the school was associated with lower rates of suicide attempts, suicidal thoughts, and self-harm. This finding is particularly important given that nearly one-third of adolescents’ report feeling unsafe at school, as seen in a cross-cultural study across 13 Asian and European countries, including India [55]. Feeling unsafe has been linked to victimization and mental health problems, including depressive symptoms and suicidal behaviour [10]. This highlights the need to create safe school environments through interventions that promote socio-emotional learning and foster positive relationships with peers and teachers [56].

#### Other factors

Urban residence, associated with increased stress and higher rates of self-harm [57], showed stronger associations in the multivariable analysis than in the univariate analysis in our study. Migration across states and using a non-native language may further increase the risk of suicidal behaviour, potentially by intensifying feelings of *thwarted belongingness* [38, 58]. In our sample, being born outside the state had a higher risk of suicide attempts in the multivariable analysis, although this association was not observed in the univariate analysis. Interestingly, having a mother tongue other than Karnataka’s commonly spoken languages (Kannada, Tulu, or Konkani) was associated with lower rates of suicide attempts in the multivariable analysis, whereas no such association was observed in the univariate analysis. Moreover, adolescents with Konkani as their native language had lower suicide attempts compared with Kannada speakers, possibly reflecting a relatively better socioeconomic status in this group. Additionally, having both parents from outside the state and not residing with biological parents were associated with a higher risk of suicidal ideation; these associations were observed only in the multivariable analysis. These exploratory associations in the univariate analysis changed after multivariable adjustment and should be considered preliminary, requiring further investigation.

### Limitations

However, several limitations should be considered. The primary limitation was a higher attrition rate in the 2023 sample (25%) compared to 2016 (7%), mainly due to student absences on the day of data collection, which may hav e introduced selection bias. Nevertheless, overall response rates in both samples remained high. Another major limitation is the limited representativeness of the sample, drawn exclusively from schools in Udupi district—a region with higher literacy, a favourable sex ratio, a strong service sector, and a higher per capita income than the national average. Adolescents from minority or socioeconomically disadvantaged background— more prevalent in northern Karnataka and at higher risk for suicidality [59], were likely underrepresented. Nevertheless, no nationally representative Indian study has examined temporal changes in adolescent suicidality, making these findings valuable even if not nationally generalizable. Future studies should include more diverse samples to improve generalizability.

Additionally, the study did not distinguish between occasional and recurrent suicidal thoughts. However, data were collected on suicide attempts, suicidal ideation with or without self-harm, and self-harm without suicide intent —clinically relevant markers of suicide risk. The assessment of self-harm may have underestimated prevalence, as only examples such as “cutting or burning the skin” were provided. Prior research suggests that broader definitions typically yield higher estimates [60, 61]. Finally, not all known risk factors—such as academic stress, substance use, and economic hardship—were assessed, as highlighted in previous reviews [45], as the study also evaluated several other domains of adolescent well-being; thus, residual confounding from unmeasured stressors cannot be ruled out.

## Conclusions

The study found increased odds of suicide attempts, ideation, and self-harm among Indian adolescents’ after pandemic, linked to mental health issues and bullying, while school safety was protective. These findings have clinical, societal, and policy implications. Effective suicide prevention should address underlying risk factors—mental health challenges, bullying, and school safety. Anti-bullying programs, still limited or poorly enforced in Indian schools [62], along with life skills training and multi-component school-based interventions, may help reduce risk [63–66]. Early identification and intervention for internalizing and externalizing problems is also crucial [67–70]. Furthermore, our findings are relevant in the context of the guidelines issued by the Supreme Court of India, which direct educational institutions to strengthen mental health safeguards for students, including access to qualified mental health professionals, staff training, written referral protocols, and reporting and monitoring mechanisms, in alignment with the National Suicide Prevention Strategy [71].

## Supplementary Information

Below is the link to the electronic supplementary material.

Supplementary Material 1: Table S1. Univariate logistic regression of risk factors for self-harm, suicidal ideations, and suicide attempts.

## Data Availability

Data is available from authors on request.
