# Supplementary material for: Changes in suicide attempt, suicidal ideation, and self-harm among Indian adolescents: comparison of cross-sectional surveys before (2016) and after (2023) COVID-19 pandemic
Source: Child Adolesc Psychiatry Ment Health. 2026 Feb 13;20:39. doi: 10.1186/s13034-026-01041-4 (PMC13005413; doi:10.1186/s13034-026-01041-4)
Supplement: Supplementary file 1 — Supplementary Material 1: Table S1. Univariate logistic regression of risk factors for self-harm, suicidal ideations, and suicide attempts. [file 13034_2026_1041_MOESM1_ESM.docx]

**Supplementary Table S1: Univariate logistic regression with all risk factors, for self-harm, suicidal ideation, and suicide attempt**

|  | **Self-harm** | | **Suicidal ideation** | | **Suicide attempt** | |
| --- | --- | --- | --- | --- | --- | --- |
|  | **n(%)** | **OR(95% CI)** | **n(%)** | **OR(95% CI)** | **n(%)** | **OR(95% CI)** |
| **Year** |  |  |  |  |  |  |
| 2016 | 123(8.5) | 1 | 67(4.7) | 1 | 30(2.1) | 1 |
| 2023 | 158(15.2) | 1.92(1.49–2.46)*** | 121(12.0) | 2.67(1.96–3.65)*** | 68(6.6) | 3.26(2.11–5.05)*** |
| **Gender** |  |  |  |  |  |  |
| Females | 129(9.7) | 1 | 90(6.8) | 1 | 46(3.5) | 1 |
| Males | 152(13.3) | 1.43(1.11–1.83)** | 98(8.6) | 1.30(0.96–1.75) | 52(4.6) | 1.33(0.89–2.00) |
| **Age** |  |  |  |  |  |  |
| ≤13 | 145(10.8) | 1 | 85(6.3) | 1 | 42(3.1) | 1 |
| >13 | 136(12.0) | 1.13(0.88–1.44) | 103(9.1) | 1.48(1.10–2.00)* | 56(5.0) | 1.62(1.08–2.44)* |
| **Place of residence** |  |  |  |  |  |  |
| Rural | 47(8.3) | 1 | 43(7.6) | 1 | 23(4.1) | 1 |
| Urban | 234(12.2) | 1.54(1.11–2.14)* | 145(7.6) | 1.00(0.70–1.42) | 75(4.0) | 0.96(0.60–1.55) |
| **Place of birth** |  |  |  |  |  |  |
| Outside of Karnataka | 2(3.5) | 1 | 3(5.3) | 1 | 2(3.6) | 1 |
| Karnataka | 278(11.5) | 3.57(0.87–14.71) | 185(7.7) | 1.50(0.46–4.84) | 95(4.0) | 1.11(0.27–4.64) |
| **Native language** |  |  |  |  |  |  |
| Kannada | 115(9.9) | 1 | 83(7.2) | 1 | 48(4.2) | 1 |
| Konkani | 24(9.0) | 0.90(0.57–1.42) | 17(6.4) | 0.88(0.51–1.51) | 5(1.9) | 0.44(0.17–1.11) |
| Tulu | 97(13.6) | 1.43(1.07–1.90)* | 66(9.3) | 1.32(0.94–1.85) | 35(4.9) | 1.18(0.76–1.85) |
| Other | 44(15.3) | 1.65(1.13–2.40)** | 22(7.7) | 1.07(0.66–1.75) | 10(3.5) | 0.84(0.42–1.68) |
| **Parents background** |  |  |  |  |  |  |
| Both from Karnataka | 268(11.5) | 1 | 175(7.6) | 1 | 93(4.0) | 1 |
| Only one from Karnataka | 6(9.8) | 0.84(0.36–1.97) | 4(6.6) | 0.86(0.31–2.40) | 2(3.3) | 0.81(0.19–3.35) |
| Both not from Karnataka | 1(3.1) | 0.25(0.03–1.82) | 4(13.0) | 1.75(0.61–5.04) | 0 | — |
| **Family structure** |  |  |  |  |  |  |
| Two biological parents | 251(10.9) | 1 | 163(7.1) | 1 | 83(3.6) | 1 |
| One biological parent | 17(16.2) | 1.58(0.92–2.70) | 13(12.0) | 1.85(1.01–3.37)* | 8(7.6) | 2.19(1.03–4.64)* |
| Other | 8(17.8) | 1.77(0.81–3.84) | 7(16.0) | 2.47(1.08–5.63)* | 4(9.3) | 2.72(0.95–7.78) |
| **Family structure +** |  |  |  |  |  |  |
| Nuclear family | 237(10.7) | 1 | 150(6.8) | 1 | 78(3.6) | 1 |
| Extended family | 14(15.9) | 1.58(0.88–2.84) | 12(14.0) | 2.19(1.16–4.12)* | 5(5.8) | 1.65(0.65–4.19) |
| Single parent | 15(15.8) | 1.56(0.89–2.76) | 12(13.0) | 1.98(1.06–3.70)* | 6(6.3) | 1.83(0.78–4.30) |
| Only relatives | 6(18.8) | 1.92(0.78–4.72) | 3(9.4) | 1.42(0.43–4.70) | 4(13.0) | 4.02(1.37–11.75)* |
| Biological parents and stepparent | 1(25.0) | 2.78(0.29–26.83) | 0 | - | 0 | — |
| Adoptive | 0 | - | 2(50.0) | 13.68(1.91–97.80)** | 0 | — |
| Other | 4(18.2) | 1.85(0.62–5.52) | 4(19.0) | 3.22(1.07–9.69)* | 2(9.5) | 2.85(0.65–12.46) |
| **Traditional bullying in school** |  |  |  |  |  |  |
| Not at all | 166(8.6) | 1 | 104(5.4) | 1 | 52(2.7) | 1 |
| Less than once a week | 67(20.4) | 2.73(2.00–3.73)*** | 39(12.0) | 2.38(1.61–3.51)*** | 20(6.2) | 2.35(1.38–3.99)** |
| More than once a week | 42(26.1) | 3.75(2.55–5.52)*** | 42(27.0) | 6.34(4.23–9.50)*** | 24(15.0) | 6.47(3.86–10.82)*** |
| **Traditional bullying outside of school** |  |  |  |  |  |  |
| Not at all | 195(9.0) | 1 | 128(5.9) | 1 | 61(2.8) | 1 |
| Less than once a week | 51(30.9) | 4.53(3.15–6.50)*** | 36(22.0) | 4.36(2.90–6.57)*** | 23(14.0) | 5.46(3.29–9.09)*** |
| More than once a week | 30(38.0) | 6.20(3.84–9.99)*** | 22(29.0) | 6.35(3.75–10.74)*** | 13(17.0) | 6.95(3.63–13.29)*** |
| **Cyberbullying** |  |  |  |  |  |  |
| Not at all | 229(10.2) | 1 | 148(6.6) | 1 | 71(3.2) | 1 |
| Less than once a week | 27(40.3) | 5.95(3.58–9.88)*** | 21(31.0) | 6.44(3.75–11.08)*** | 15(22.0) | 8.76(4.71–16.30)*** |
| More than once a week | 13(28.3) | 3.47(1.80–6.69)*** | 10(22) | 4.03(1.96–8.30)*** | 9(20.0) | 7.39(3.43–15.89)*** |
| **School safety** |  |  |  |  |  |  |
| Never feel safe | 39(24.4) | 1 | 29(19.0) | 1 | 24(15) | 1 |
| Sometimes feel safe | 71(16.1) | 0.59(0.38–0.92)* | 48(11.0) | 0.54(0.33–0.90)* | 22(5.0) | 0.29(0.16–0.54)*** |
| Often feel safe | 29(12.8) | 0.46(0.27–0.78)** | 11(4.9) | 0.23(0.11–0.47)*** | 6(2.7) | 0.15(0.06–0.38)*** |
| Always feel safe | 137(8.5) | 0.29(0.19–0.43)*** | 97(6.0) | 0.28(0.18–0.45)*** | 44(2.8) | 0.16(0.09–0.27)*** |
| **Health problems** |  |  |  |  |  |  |
| No | 221(10.4) | 1 | 141(6.7) | 1 | 69(3.3) | 1 |
| Yes | 52(16.9) | 1.75(1.26–2.43)** | 39(13) | 2.05(1.41–2.99)*** | 25(8.2) | 2.62(1.63–4.21)*** |
| **SDQ**^†^ | **Mean(SD)** |  | **Mean(SD)** |  | **Mean(SD)** |  |
| Emotion problems | 2.9(2.2) | 1.21(1.15–1.28)*** | 3.0(2.2) | 1.33(1.25–1.42)*** | 3.0(2.2) | 1.26(1.16–1.37)*** |
| Hyperactivity | 2.8(1.9) | 1.21(1.14–1.29)*** | 2.8(1.9) | 1.31(1.22–1.41)*** | 2.8(1.9) | 1.26(1.14–1.39)*** |
| Conduct problems | 2.8(1.7) | 1.34(1.25–1.43)*** | 2.8(1.7) | 1.49(1.37–1.61)*** | 2.8(1.7) | 1.51(1.36–1.68)*** |
| Peer problems | 2.6(1.7) | 1.19(1.11–1.27)*** | 2.6(1.7) | 1.23(1.13–1.33)*** | 2.6(1.7) | 1.27(1.14–1.41)*** |
| Prosocial behavior | 7.4(2.1) | 0.95(0.90–1.00) | 7.4(2.2) | 0.96(0.90–1.03) | 7.4(2.2) | 0.82(0.75–0.89)*** |

SDQ: Strengths and Difficulties Questionnaire; ^†^Odds calculated for 1 SD change; *p<.05, **p<.01, ***p<.001
